# Molecular Spectrum of Autosomal Dominant Hypercholesterolemia in France

**DOI:** 10.1002/humu.21348

**Published:** 2010-11

**Authors:** Marie Marduel, Alain Carrié, Agnes Sassolas, Martine Devillers, Valérie Carreau, Mathilde Di Filippo, Danièle Erlich, Marianne Abifadel, Alice Marques-Pinheiro, Arnold Munnich, Claudine Junien, Catherine Boileau, Mathilde Varret, Jean-Pierre Rabès, The French ADH Research Network

**Affiliations:** Institut National de la Santé et de la Recherche MédicaleU781, 75015, Paris, France; Université Paris Descartes75006, Paris, France; Service de Biochimie Endocrinienne et OncologiqueGH Pitié- Salpêtrière (AP-HP), 75005, Paris, France; UF Dyslipidémies-Cardiobiologie, CBPE, Hospices Civils de Lyon (GHE)Bron, France; Service d'Endocrinologie-MétabolismeGH Pitié-Salpétrière, 75005, Paris, France; Laboratoire de Biochimie et de Biologie MoléculaireCHU Saint-Louis (AP-HP & Université Paris Denis Diderot), 75019, Paris, France; Faculté de Pharmacie, Université Saint-JosephBeirut, Lebanon; AP-HP, Hôpital Ambroise Paré, Service de Biochimie et Génétique MoléculaireBoulogne, 92104, France; Université Versailles Saint-Quentin-en-Yvelines, UFR de Médecine Paris Ile-de-France OuestGuyancourt, 78280, France

**Keywords:** Autosomal Dominant Hypercholesterolemia, Mutation screening, French population, Genotype/phenotype correlation

## Abstract

Autosomal Dominant Hypercholesterolemia (ADH), characterized by isolated elevation of plasmatic LDL cholesterol and premature cardiovascular complications, is associated with mutations in 3 major genes: *LDLR* (LDL receptor), *APOB* (apolipoprotein B) and *PCSK9* (proprotein convertase subtilisin-kexin type 9). Through the French ADH Research Network, we collected molecular data from 1358 French probands from eleven different regions in France. Mutations in the *LDLR* gene were identified in 1003 subjects representing 391 unique events with 46.0% missense, 14.6% frameshift, 13.6% splice, and 11.3% nonsense mutations, 9.7% major rearrangements, 3.8% small in frame deletions/insertions, and 1.0% UTR mutations. Interestingly, 175 are novel mutational events and represent 45% of the unique events we identified, highlighting a specificity of the LDLR mutation spectrum in France. Furthermore, mutations in the *APOB* gene were identified in 89 probands and in the *PCSK9* gene in 10 probands. Comparison of available clinical and biochemical data showed a gradient of severity for ADH-causing mutations: FH=PCSK9>FDB>‘Others’ genes. The respective contribution of each known gene to ADH in this French cohort is: *LDLR* 73.9%, *APOB* 6.6%, PCSK9 0.7%. Finally, in 19.0% of the probands, no mutation was found, thus underscoring the existence of ADH mutations located in still unknown genes. © 2010 Wiley-Liss, Inc.

## INTRODUCTION

Hypercholesterolemia is a major risk factor for atherosclerosis and its premature cardiovascular complications. Hypercholesterolemia can be multifactorial or less frequently monogenic, leading to Autosomal Dominant Hypercholesterolemia (ADH; MIM# 143890) characterized by an elevation of plasmatic LDL cholesterol levels and xanthoma, xanthelasma, arcus corneae or premature coronary heart disease. The diagnosis of ADH is difficult, due to the overlap of cholesterol values between monogenic and multifactorial forms. DNA testing provides an unequivocal diagnosis and allows the identification of affected relatives at an early age when they can be offered lifestyle advice and appropriate lipid-lowering therapies (Humphries et al. 2008).

The first ADH causative gene identified was *LDLR* encoding the LDL receptor (Goldstein et al. 1973). This disease was named FH for Familial Hypercholesterolemia (MIM# 606945) and its heterozygous prevalence was estimated at 1/500. To date, over 1000 mutations in *LDLR* have been implicated in ADH (Villéger et al. 2002; Leigh et al. 2008). Subsequently, a second gene was involved after the discovery of hypercholesterolemic patients with normal LDL receptor activity (Innerarity et al. 1987). They carried a missense mutation (p.Arg3527Gln previously named p.Arg3500Gln) in *APOB*, encoding apolipoprotein B, the main ligand for the LDL receptor (Soria et al. 1989). This new molecular disorder was called FDB for Familial Defective apolipoprotein B-100 (MIM# 144010) and its frequency has been estimated at 1/250 in Switzerland and 1/1250 in Northern Europe and the US (Rabès et al. 2000). Subsequently, we identified a third ADH-causative gene: proprotein convertase subtilisin-kexin type 9 (*PCSK9*; MIM# 607787) (Abifadel et al. 2003). PCSK9 has been shown to degrade LDL receptor independently of its catalytic activity (McNutt et al. 2007). Very recently, we mapped a fourth major locus for ADH at 16q22.1 that we named *HCHOLA4* (Marques-Pinheiro et al. 2010). Finally, the proportion of ADH patients for whom the disease is not explained by a mutation in, either, *LDLR, APOB*, or *PCSK9* was estimated at 15.25 % (Varret et al. 2008). The aim of this study was to assess the molecular epidemiology of ADH in a representative French population.

## MATERIALS AND METHODS

### Proband and family recruitment

ADH probands and families were recruited by the French National Research Network on Hypercholesterolemia that includes numerous clinicians from different cities in France. Since 2005, they selected probands meeting the following inclusion criteria: total and LDL-cholesterol levels above the 95 ^th^ percentile when compared with a sex-and age-matched French population (STANISLAS cohort, B. Herbeth, G. Siest & S Visvikis-Siest, personal communication; Siest et al. 1998), autosomal dominant transmission of hypercholesterolemia in the family. Venous blood samples were sent to 3 genetic laboratories certified for ADH molecular diagnosis (A.S., A.C. & JP.R.) where DNA was extracted. The number of probands included (1358) and the diversity of their geographical origin (11 different French regions), constitute a representative sample of the French population. The study was performed in accordance with French bioethics regulations and all subjects gave informed consent.

### Candidate gene analysis

The *APOB*-p.Arg3527Gln mutation was detected as previously described (Rabès et al. 1997) or by sequencing (NM_000384.2). The promoters, the 18 exons of *LDLR* (NM_000527.3), and the 12 exons of *PCSK9* (NM_174936.3), as well as close flanking intronic sequences, were amplified. Primer sequences and annealing temperatures are available on request. Electrophoregrams were analyzed using Gensearch®, a DNA sequence analysis software developed by PhenoSystems SA, Belgium (http://www.phenosystems.com). Detection of deletions/duplications of one or more exons of *LDLR* was performed with SALSA MLPA kit (P062) and data were analyzed with Coffalyser software (MRC-Holland). In all subjects, genes were studied sequentially: at first, the *APOB*-p.Arg3527Gln mutation was looked for and the *LDLR* gene was sequenced. If no mutation was found, the search for a deletion/duplication of *LDLR* was performed. Finally, if no deletion/duplication was discovered, the *PCSK9* gene was sequenced.

### Nomenclature

All existing and new mutations were described following the recommendations of the Human Genome Variation Society at http://www.hgvs.org/mutnomen. Nucleotide numbering reflects cDNA numbering with +1 corresponding to the A of the ATG translation initiation codon in the reference sequence. Furthermore, amino acid variants now follow the standard nomenclature with the initiating methionine given as number one, rather than the historical numbering from the first residue of the mature peptide. Hence, 21 or 27 has been added to all original amino acid numbering for LDL receptor or apo B, respectively. Variants in the 5′ untranslated region are now numbered from the nucleotide immediately preceding the A of the initiating methionine.

### *In silico* prediction of effect of molecular event on LDL receptor

The causal effect of each new molecular event was estimated with *in silico* prediction of protein function using the following tools: NetGene2 (http://www.cbs.dtu.dk/services/NetGene2), NNSPLICE (http://www.fruitfly.org/seq_tools/splice.html), Polyphen (http://genetics.bwh.harvard.edu/pph), SIFT (http://sift.jcvi.org), Pmut (http://mmb2.pcb.ub.es:8080/PMut) and SNP3D (http://www.snps3d.org). The reference sequences used for LDLR were P01130.1 (SwissProt) orNP_000518.1 (NCBI RefSeq).

### Statistical analysis

**Supp. Figure S1:**
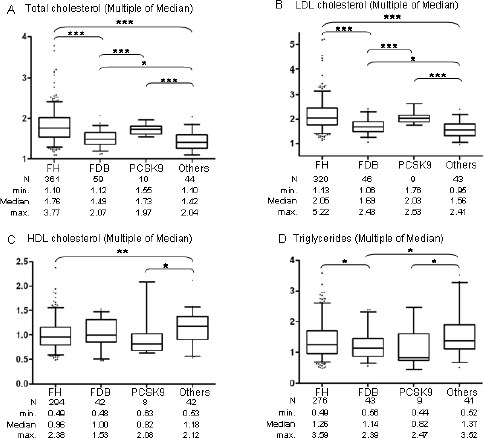
Comparison of lipid levels among the four molecular groups of patients. Panel **A**: Multiple of Median (MoM) for Total cholesterol levels. Panel **B**: Multiple of Median (MoM) for LDL cholesterol levels. Panel **C**: Multiple of Median (MoM) for HDL cholesterol levels. Panel **D**: Multiple of Median (MoM) for triglycerides levels. Results are presented with the median and range from minimum to maximum MoM values for each group. Median Mann-Whitney Test: * p < 0.05, ** p < 0.01, *** p < 0.001.

When possible, we collected clinical and/or biochemical data under fasting conditions and without any cholesterol lowering drug. Plasma levels of total-, LDL-, HDL-cholesterol, triglycerides, and clinical signs of the disease were not available for all probands, thus sample size is different among each lipid parameter as presented in Supp. Figure S1. Lipid levels were expressed as multiples of median (MoM) for age and gender of a reference French population: the STANISLAS cohort. Comparison of quantitative values (lipid levels and age) was performed by the Mann-Whitney test with Graph Pad Prism 5.03 software. Results are presented with the median and range from minimum to maximum MoM values. Comparison of qualitative values was performed with the Chi-Square Test (or Fisher Test for N<5) online with the StatPages at http://www.statpages.org.

## RESULTS AND DISCUSSION

Through the ADH French Research Network, we collected molecular data from 1358 French ADH probands and found 1111 molecular events: 1012 (91.1%) *LDLR* mutations in 1003 (73.9%) probands, 9 with two *LDLR* variants each; 89 (8.0%) *APOB*-p.Arg3527Gln mutation in 89 (6.6%) probands including 2 probands also heterozygous for a *LDLR* mutation; 10 (0.9%) *PCSK9* mutations in 10 (0.7%) probands. For the 258 remaining probands (19.0%), no mutation was identified in the three major ADH genes.

### Variations in *LDLR*

Variations in *LDLR* were identified in 1003 probands representing 391 unique events distributed as follows: 46.0% missense; 11.3% nonsense; 14.6% frameshift; 3.8% small in frame deletions, insertions, or indels; 13.6% splice; 1.0% in 5′UTR; and 9.7% large deletions or duplications (complete list available on request). In accordance with the known heterogeneity of the French population, this distribution was similar to that reported worldwide (Leigh et al. 2008) (Supp. Table S1). However, splice mutations were significantly more abundant in this French cohort (p=0.002), probably indicating a technological bias. Indeed, systematic sequencing of intronic sequences is a more recent practice (Amsellem et al. 2002).

Within the 283 variations newly reported in this French population, 175 were novel mutational events (Tables 1, 2, and 3) and represent 45% (175/391) of the unique events we identified and 22% (222/1003) of probands with a variation in *LDLR* (1 with two new *LDLR* variants). Furthermore, *LDLR* mutational events newly reported in France represent 72% (283/391) of the unique events reported here and 41% (416/1003) of *LDLR* variation carriers. This highlights a higher level of allelic heterogeneity for *LDLR* and indicates a specificity of the spectrum of *LDLR* mutations in France when compared to other countries. Another method for genetic diagnosis of ADH is based on a DNA-array platform that is able to detect 242 different point mutations in *LDLR* and 3 in *APOB* (Lipochip version 8, http://www.progenika.com). The Lipochip used to screen clinically diagnosed FH patients in Spain was able to detect mutations in 78% of all carriers (Alonso R et al. 2009). If the Lipochip (version 8) had been used to screen this French cohort, it only would have detected 40% of the mutation carriers, thus indicating the need for specific national screening strategies.

**Table 1:** New mutational events leading to abnormal protein size

Location	cDNA (HGVS)	Protein (HGVS)	Predicted protein	Number of probands	Familial segregation (*)
***nonsense***					
exon 2	c.102C>A	p.Cys34X	33 AA, truncated or no protein	1	na
exon 4	c.535G>T	p.Glu179X	178 AA, truncated or no protein	1	na
exon 4	c.539G>A	p.Trp180X	179 AA, truncated or no protein	3	na
exon 10	c.1532T>G	p.Leu511X	510 AA, truncated or no protein	1	na
exon 11	c.1598G>A	p.Trp533X	532 AA, truncated or no protein	1	na
exon 11	c.1685G>A	p.Trp562X	561 AA, truncated or no protein	1	yes (3 - 1)
exon 13	c.1860G>A	p.Trp620X	619 AA, truncated or no protein	1	yes (2 - 1)
exon 14	c.1997G>A	p.Trp666X	665 AA, truncated or no protein	1	na
exon 17	c.2446A>T	p.Lys816X	815 AA, truncated or no protein	1	na
***frameshifts***					
exon 3	c.244del	p.Cys82AlafsX124	81 AA with 124 novel AA, truncated or no protein	1	na
exon 4	c.350_372dup	p.Gln125ThrfsX89	124 AA with 89 novel AA, truncated or no protein	1	na
exon 4	c.357del	p.Lys120SerfsX86	119 AA with 86 novel AA, truncated or no protein	1	na
exon 4	c.374_375insCTGA	p.Gln125HisfsX2	124 AA with 2 novel AA, truncated or no protein	1	yes (2 - 2)
exon 4	c.450dup	p.Ala151ArgfsX29	150 AA with 29 novel AA, truncated or no protein	1	na
exon 4	c.482_488del	p.Ile161SerfsX43	160 AA with 43 novel AA, truncated or no protein	1	na
exon 4	c.609del	p.Cys204AlafsX2	203 AA with 2 novel AA, truncated or no protein	1	na
exon 4	c.664_681delinsCCGACTG	p.Cys222ProfsX14	221 AAwith 14 novel AA, truncated or no protein	1	na
exon 4	c.666_687del	p.Cys222X	221 AA, truncated or no protein	1	na
exon 4	c.673_682delinsTGCAA	p.Lys225CysfsX13	224 AAwith 13 novel AA, truncated or no protein	2	na
exon 4	c.681_682insTGAG	p.Glu228X	227 AA, truncated or no protein	1	na
exon 4	c.682del	p.Glu228ArgfsX37	227 AA with 37 novel AA, truncated or no protein	2	yes (3 - 1)
exon 5	c.752dup	p.Ser252GlnfsX5	251 AA with 5 novel AA, truncated or no protein	1	yes (2 - 1)
exon 5	c.781del	p.Cys261AlafsX4	260 AA with 4 novel AA, truncated or no protein	1	na
exon 6	c.865del	p.Cys289AlafsX81	288 AA with 81 novel AA, truncated or no protein	1	na
exon 6	c.875dup	p.Asp293GlyfsX8	292 AA with 8 novel AA, truncated or no protein	2	na
exon 7	c.951del	p.Glu317AspfsX53	316 AA with 53 novel AA, truncated or no protein	1	na
exon 7	c.1008del	p.Tyr336X	335 AA, truncated or no protein	3	na
exon 7	c. 1031 del	p.Phe344SerfsX26	343 AA with 26 novel AA, truncated or no protein	3	na
exon 7	c.1042del	p.Ala348ProfsX22	347 AA with 22 novel AA, truncated or no protein	1	na
exon 8	c.1099_1104delinsGT	p.Leu367ValfsX2	366 AA with 2 novel AA, truncated or no protein	1	yes (2 - 0)
exon 9	c.1343del	p.Gln448ArgfsX3	447 AA with 3 novel AA, truncated or no protein	1	na
exon 10	c.1496_1497del	p.Ser499CysfsX36	498 AA with 36 novel AA, truncated or no protein	1	na
exon 10	c.1549_1555del	p.Ser517GlnfsX29	516 AA with 29 novel AA, truncated or no protein	1	na
exon 11	c.1610del	p.Gly537GlufsX11	536 AA with 11 novel AA, truncated or no protein	1	na
exon 11	c.1632del	p.Gly546AlafsX2	545 AA with 2 novel AA, truncated or no protein	2	yes (3 - 0)
exon 12	c.1718del	p.Gly573AlafsX92	572 AA with 92 novel AA, truncated or no protein	1	na
exon 12	c.1737del	p.Ser580ProfsX85	579 AA with 85 novel AA, truncated or no protein	4	na
exon 12	c.1749_1753del	p.Ser584LeufsX17	583 AA with 17 novel AA, truncated or no protein	1	na
exon 13	c.1886del	p.Phe629SerfsX36	628 AA with 36 novel AA, truncated or no protein	1	na
exon 13	c.1934dup	p.Asn645LysfsX24	644 AA with 24 novel AA, truncated or no protein	1	na
exon 13	c.1948_1952dup	p.Asp651GlufsX16	650 AA with 16 novel AA, truncated or no protein	1	yes (3 - 0)
exon 13	c.1961_1965dup	p.His656SerfsX11	655 AA with 11 novel AA, truncated or no protein	1	na
exon 14	c.2013_2014del	p.Leu672GlufsX44	671 AA with 44 novel AA, truncated or no protein	1	na
exon 14	c.2030_2042del	p.Cys677SerfsX28	676 AA with 28 novel AA, truncated or no protein	1	na
exon 15	c.2187_2197del	p.Lys730HisfsX48	729 AA with 48 novel AA, truncated or no protein	1	na
exon 15	c.2230del	p.Arg744AspfsX21	743 AA with 21 novel AA, truncated or no protein	1	yes (2 - 0)
exon 16	c.2318del	p.Gly773AlafsX15	772 AA with 15 novel AA, truncated or no protein	1	na
exon 17	c.2403_2406del	p.Leu802AlafsX126	801 AA with 126 novel AA, truncated or no protein	1	na
exon 17	c.2416del	p.Val806SerfsX123	805 AA with 123 novel AA, truncated or no protein	2	na
exon 17	c.2509del	p.His837ThrfsX92	836 AA with 92 novel AA, truncated or no protein	1	na
***major rearangements***		***MLPA results***	***Predicted protein if recombinaison between Alu not affecting splice sites***		
Prom	c.1-?_1060+?del	del from prom. to exon 7	no in phase ATG within exon 8, no protein	1	yes (2 - 2)
Prom	c.1-?_3428+?del	del from prom. to exon 18	no protein	1	yes (2 - 2)
exon 1	c.1-?_67+?del	del of exon 1	no in phase ATG within exon 2, no protein	2**	na
exon 1	c.1-?_3428+?del	del of exons 1 to 18	no protein	1	na
exon 2	c.68-?_817+?dup dup of exons 2 to	5	p.Gly24Val273 dup, elongated protein (249 AA)	2**	na
exon 2	c.68-?_1586+?del	del of exons 2 to 10	p.Val23AlafsX19, truncated protein	1	na
exon 2	c.68-?_1705+?del	del of exons 2 to 11	p.Val23Asp;Gly24_Asp569del, shortened protein (545 AA)	2**	na
exon 2	c.68-?_2140+?del	del of exons 2 to 14	p.Val23Glu;Gly24_Glu714del, shortened protein (690 AA)	1	na
exon 2	c.68-?_2547+?del	del of exons 2 to 17	p.Val23GlufsX9, truncated protein	1	na
exon 3	c.191-?_313+?del	del of exon 3	p.Leu64Ser;Ser65_Pro105del, shortened protein (40 AA)	1	na
exon 3	c.191-?_694+?del	del of exons 3 and 4	p.Leu64Ser;Ser65_Ala232del, shortened protein (167 AA)	1	na
exon 4	c.314-?_940+?dup	dup of exons 4 to 6	p.Gly314Ala;Pro106_Cys313 dup, elongated protein (207 AA)	1	yes (3 - 1)
exon 5	c. 695-?_1586+?del	del of exons 5 to 10	p.Ala233ValfsX18, truncated protein	1	na
exon 9	c.1187-?_3428+?del	del of exons 9 to 18	no protein	2**	na
exon 11	c.1586-?_1705+?del	del of exon 11	p.Phe530SerfsX10, truncated protein	2**	yes (3 - 3), na
exon 12	c.1706-?_1845+?dup	dup of exon 12	p.Asp616Ile fsX96, truncated protein	1	na
exon 12	c.1706-?_2389+?del	del of exons 12 to 16	p.Asp569Val;Leu570_Val797del, shortened protein (227 AA)	3**	na
exon 13	c.1846-?_2140+?dup	dup of exons 13 and 14	p.Glu714GlyfsX29, truncated protein	4*	na
1 and 8	c.1-?_190+?del 1061?_1845+?del	del of exons 1-2 and 8 to 12	no in phase ATG within exon 3, no protein	2**	na

na: not available.

* nb of affected carriers - nb of unaffected non carriers.

** all unrelated carriers may present different events since the exact breakpoints of these major rearrangements are unknown.

**Table 2:** New intronic and in frame deletion or insertion variations

Location	cDNA (HGVS)	Protein (HGVS)	Number of probands	Familial segregation (*)	Splice modification prediction		
					NetGene2	NNSPLICE	Conclusion
***intronic events***							
intron 4	c.693_694+20del		1	na	new DS at +60	new DS at +60	deleterious
intron 4	c.694+1G>T		1	na	loss of DS	loss of DS	deleterious
intron 6	c.940+14del		1	na	loss of DS	loss of DS	deleterious
intron 6	c.940+1G>A		1	na	loss of DS	loss of DS	deleterious
intron 6	c.940+1G>C		1	na	loss of DS	loss of DS	deleterious
intron 6	c.940+2T>A		1	na	loss of DS	loss of DS	deleterious
Intron 6	c.941-12G>A#		1	yes (3 - 0)	no change	no change	benign
intron 7	c.1060+24C>A		1	na	new DS at +11	no change	?
intron 7	c.1060+26 T>G		1	na	no change	no change	benign
intron 8	c.1187-1G>A		1	na	loss of AS	na	?
intron 9	c.1358+3_1358+8del		1	yes (7 - 8)	loss of DS	loss of DS	deleterious
intron 9	c.1359-4T>C		1	na	no change	no change	benign
intron 9	c.1359-25T>A		1	na	no change	no change	benign
intron 10	c.1587-2A>T		2	yes (2 - 2)	loss of AS	loss of AS	deleterious
intron 11	c.1705+2_+3insC		1	na	loss of DS	loss of DS	deleterious
intron 11	c.1706-2A>T		1	na	new AS at 1715	na	?
intron 11	c.1706-24T>C		1	na	no change	no change	benign
intron 15	c.2311+1G>T		2	yes (4 - 2)	loss of DS	loss of DS	deleterious
intron 16	c.2389+14G>A		1	na	no change	no change	benign
intron 17	c.2547+5G>C		1	na	no change	new DS at +114	?
***in frame deletions or insertions***							
exon 4	c.316_336del	p.Pro106_Asp112del	2	yes (3 - 1)	no change	no change	benign
exon 4	c.516_524dup	p.Cys173_Asp175dup	1	yes (2 - 0)	no change	no change	benign
exon 4	c.648_656del	p.Asp217_Gly219del	1	na	no change	no change	benign
exon 4	c.667_693del	p.Lys223_Cys231del	1	na	no change	new DS at +59	?
exon 4	c.673_681dup	p.Lys225_Asp227dup	1	na	no change	no change	benign
exon 4	c.682_683insAAATCTGAC	p.Asp227_Glu228InsLysSerAsp	1	na	no change	no change	benign
exon 7	c.964_966del	p.Asn322del	1	na	no change	no change	benign
exon 11	c.1629_1652del	p.Lys543_Asp551delinsAsn	1	na	no change	no change	benign
exon 12	c.1730_1738del	p.Trp577_Asp579del	1	na	no change	no change	benign
exon 12	c.1829_1831del	p.Ser610del	2	yes (5 - 1)	no change	no change	benign
***exonic events***							
exon 9	c.1194C>T	p.Ile398Ile	1	na	no change	no change	benign
exon 12	c.1813C>T	p.Leu605Leu	1	yes (4 - 1)	new DS at 1813	new DS at 1813	deleterious
exon 14	c.2140G>C	p.Glu714Gln	1	na	loss of DS	loss of DS	deleterious

Splice modification predicted with NetGene2 (http://www.cbs.dtu.dk/services/NetGene2) and NNSPLICE (http://www.fruitfly.org/seq_tools/splice.html) softwares.

na: not available.

* nb of affected carriers - nb of unaffected non carriers. DS: donor site. AS: Acceptor site.

# Variation effect tested by RT-PCR.

The reference sequences used for*LDLR* were P01130.1 (SwissProt) or NP_000518.1 (NCBI RefSeq).

**Table 3:** New missense variations

					Prediction of damaging effect at the protein level						
					
Loca-tion	cDNA (HGVS)	Protein (HGVS)	Number of probands	FSA(*)	Polyphen			SIFT	Pmut	SNPs3D, deleterious (SVM score)	Conclusion
									
					structural effect	protein domain	damaging				
exon 2	c.100T>G	p.Cys34Gly	1	yes (3-0)	S-S bond disrupted	Extracell.	probably	Not Tolerated	Neutral	yes (-3.80)	deleterious
exon 3	c.233G>A	p.Arg78His	1	na		Extracell.	benign	Tolerated	Neutral	no (0.74)	benign
exon 3	c.244T>G	p.Cys82Gly	3	na	S-S bond disrupted	Extracell.	probably	Not Tolerated	Pathological	yes (-3.75)	deleterious
exon 3	c.255G>T	p.Gln85His	1	na		Extracell.	benign	Tolerated	Neutral	no (1.36)	benign
exon 3	c.265T>G	p.Cys89Gly	1	na	S-S bond disrupted	Extracell.	probably	Not Tolerated	Pathological	yes (-3.75)	deleterious
exon 3	c.270T>A	p.Asp90Glu	4	yes (4-3, 3-1), na	LB site disrupted	Extracell.	probably	Not Tolerated	Neutral	yes (-2.04)	deleterious
exon 3	c.291C>G	p.Asn97Lys	1	na	Close to LB site	Extracell.	possibly	Not Tolerated	Neutral	yes (-1.11)	deleterious
exon 3	c.310T>C	p.Cys104Arg	1	na	S-S bond disrupted	Extracell.	probably	Not Tolerated	Pathological	yes (-3.26)	deleterious
exon 4	c.362G>A	p.Cys121Tyr	1	na	S-S bond disrupted	Extracell.	probably	Not Tolerated	Pathological	yes (-3.18)	deleterious
exon 4	c.382T>C	p.Cys128Arg	1	na	S-S bond disrupted	Extracell.	probably	Not Tolerated	Pathological	yes (-4.21)	deleterious
exon 4	c.383G>T	p.Cys128Phe	1	na	S-S bond disrupted	Extracell.	probably	Not Tolerated	Pathological	yes (-3.86)	deleterious
exon 4	c.416A>T	p.Asp139Val	1	yes (2-2)	LB site disrupted	Extracell.	probably	Not Tolerated	Neutral	yes (-3.82)	deleterious
exon 4	c.416A>G	p.Asp139Gly	1	na	LB site disrupted	Extracell.	probably	Not Tolerated	Neutral	yes (-2.10)	deleterious
exon 4	c.427T>G	p.Cys143Gly	1	yes (4-2)	S-S bond disrupted	Extracell.	probably	Not Tolerated	Neutral	yes (-2.70)	deleterious
					S-S bond						
exon 4	c.464G>A	p.Cys155Tyr	1	na	disrupted, HdpC and Overpacking at BS	Extracell.	probably	Not Tolerated	Pathological	yes (-2.62)	deleterious
exon 4	c.501C>G	p.Cys167Trp	3	na	S-S bond disrupted	Extracell.	probably	Not Tolerated	Pathological	yes (-3.99)	deleterious
exon 4	c.589T>G	p.Cys197Gly	1	na	S-S bond disrupted	Extracell.	probably	Not Tolerated	Neutral	yes (-3.47)	deleterious
exon 4	c.598T>A	p.Phe200lle	1	na		Extracell.	benign	Tolerated	Neutral	no (1.38)	benign
exon 4	c.611G>A	p.Cys204Tyr	1	na	S-S bond disrupted	Extracell.	probably	Not Tolerated	Pathological	yes (-3.06)	deleterious
exon 4	c.641G>C	p.Trp214Ser	1	na	Close to LB site	Extracell.	probably	Tolerated	Pathological	yes (-2.05)	deleterious
exon 4	c.669G>C	p.Lys223Asn	2	na		Extracell.	benign	Tolerated	Neutral	no (1.11)	benign
exon 4	c.680A>T	p.Asp227Val	1	yes (4-1)	LB site disrupted	Extracell.	probably	Not Tolerated	Neutral	yes (-3.18)	deleterious
exon 4	c.693C>G	p.Cys231Trp	1	na	S-S bond disrupted	Extracell.	probably	Not Tolerated	Pathological	yes (-3.71)	deleterious
exon 5	c.793A>T	p.Ser265Cys	1	na		Extracell.	probably	Not Tolerated	Neutral	yes (-2.03)	deleterious
exon 6	c.869T>G	p.Ile290Ser	1	na	Close to LB site	Extracell.	probably	Not Tolerated	Neutral	yes (-1.95)	deleterious
exon 6	c.887G>A	p.Cys296Tyr	1	na	S-S bond disrupted	Extracell.	probably	Not Tolerated	Pathological	yes (-1.81)	deleterious
exon 6	c.914G>C	p.Trp305Ser	1	na		Extracell.	probably	Not Tolerated	Neutral	possibly (-0.10)	deleterious
exon 7	c.965A>T	p.Asn322lle	1	na		Extracell.	probably	Not Tolerated	Neutral	yes (-1.41)	deleterious
exon 7	c.1007A>G	p.Tyr336Cys	1	na	Close to LB site	Extracell.	probably	Tolerated	Neutral	possibly not (0.13)	benign
exon 7	c.1019 1020delins TG	p.Cys340Leu	1	na	S-S bond disrupted	Extracell.	probably	Not Tolerated	Neutral	yes (-3.27)	deleterious
exon 7	c.1055G>T	p.Cys352Phe	1	na	S-S bond disrupted	Extracell.	probably	Not Tolerated	Pathological	yes (-3.27)	deleterious
exon 8	c.1061A>C	p.Asp354Ala	1	na	LB site disrupted, HdpC and CC at BS	Extracell.	probably	Not Tolerated	Neutral	yes (-2.25)	deleterious
exon 8	c.1067A>C	p.Asp356Ala	1	la	Close to LB site	Extracell.	probably	Not Tolerated	Pathological	yes (-1.58)	deleterious
exon 8	c.1103G>C	p.Cys368Ser	1	na	S-S bond disrupted	Extracell.	probably	Not Tolerated	Neutral	yes (-3.27)	deleterious
exon 8	c.1153C>G	p.Leu385Val	1	na		Extracell.	benign	Tolerated	Neutral	possibly (-0.23)	benign
exon 9	c.1223A>C	p.Glu408Ala	2	na		YWTD-EGF	possibly	Tolerated	Neutral	possibly (-0.47)	?
exon 9	c.1288G>C	p.Val430Leu	1	na		YWTD-EGF	possibly	Tolerated	Neutral	yes (-1.19)	?
exon10	c.1424C>T	p.Ala475Val	2	na		YWTD-EGF	benign	Tolerated	Neutral	no (0.60)	benign
exon10	c.1460A>G	p.Asn487Ser	1	na		YWTD-EGF	probably	Tolerated	Neutral	yes (-0.90)	?
exon 10	c.1487G>T	p.Gly496Val	1	na	Overpacking at BS	YWTD-EGF	probably	Tolerated	Neutral	possibly (-0.37)	?
exon 10	c.1519A>G	p.Lys507Glu	1	na		YWTD-EGF	benign	Tolerated	Neutral	possibly not (0.12)	benign
exon 10	c.1546G>A	p.Gly516Ser	1	na		YWTD-EGF	benign	Tolerated	Neutral	yes (-1.93)	benign
exon 10	c.1567G>T	p.Val523Leu	1	na		YWTD-EGF	possibly	Not Tolerated	Neutral	yes (-2.47)	deleterious
exon 10	c.1577C>G	p.Pro526Arg	1	na	Overpacking and CC at BS	YWTD-EGF	probably	Not Tolerated	Neutral	yes (-2.63)	deleterious
exon 11	c.1597T>C	p.Trp533Arg	1	na	CC and HdpC at BS	YWTD-EGF	probably	Not Tolerated	Pathological	yes (-4.04)	deleterious
exon 11	c.1606T>G	p.Trp536Gly	1	na		YWTD-EGF	probably	Tolerated	Pathological	yes (-3.12)	deleterious
exon11	c.1625T>G	p.Ile542Ser	1	na	HdpC and Cavity creation at BS	YWTD-EGF	probably	Not Tolerated	Pathological	yes (-4.06)	deleterious
exon 11	c.1633G>A	p.Gly545Arg	2	na	Overpacking at BS	YWTD-EGF	probably	Not Tolerated	Pathological	yes (-1.73)	deleterious
exon 11	c.1644T>G	p.Asn548Lys	2	yes (3-1)		YWTD-EGF	probably	Not Tolerated	Neutral	yes (-2.06)	deleterious
exon11	C.1687OT	p.Pro563Ser	1	na		YWTD-EGF	probably	Not Tolerated	Neutral	yes (-3.18)	deleterious
exon11	c.1703T>C	p.Leu568Pro	1	na		YWTD-EGF	probably	Not Tolerated	Neutral	yes (-3.01)	deleterious
exon 11	c.1705G>T	p.Asp569Tyr	1	na		YWTD-EGF	probably	Not Tolerated	Pathological	yes (-4.15)	deleterious
exon 12	c.1727A>C	p.Tyr576Ser	1	na	New cavity at BS	YWTD-EGF	probably	Not Tolerated	Neutral	yes (-3.17)	deleterious
exon 12	c.1736A>G	p.Asp579Gly	1	na		YWTD-EGF	probably	Not Tolerated	Pathological	yes (-3.53)	deleterious
exon 12	c.1793T>C	p.Ile598Thr	1	yes (2-1)		YWTD-EGF	possibly	Not Tolerated	Neutral	yes (-1.68)	deleterious
exon 12	c.1844A>T	p.Glu615Val	3	yes (5-8)		YWTD-EGF	probably	Not Tolerated	Neutral	yes (-3.48)	deleterious
exon 13	c.1853T>G	p.Val618Gly	1	na		YWTD-EGF	probably	Not Tolerated	Neutral	yes (-2.90)	deleterious
exon13	c.1856T>C	p.Phe619Ser	2	na		YWTD-EGF	probably	Not Tolerated	Neutral	yes (-2.90)	deleterious
exon 13	c.1907G>A	p.Gly636Asp	1	na		YWTD-EGF	probably	Not Tolerated	Neutral	yes (-3.01)	deleterious
exon13	c.1928C>T	p.Ala643Val	1	na		YWTD-EGF	benign	Tolerated	Pathological	no (1.55)	benign
exon 13	C.1945OT	p.Pro649Ser	1	yes (2-0)		YWTD-EGF	probably	Tolerated	Neutral	yes (-2.16)	?
exon 13	c.1955T>C	p.Met652Thr	1	na		YWTD-EGF	probably	Not Tolerated	Neutral	yes (-1.46)	deleterious
exon 13	c.1958T>G	p.Val653Gly	1	na		YWTD-EGF	probably	Tolerated	Neutral	possibly (-0.45)	?
exon 13	c.1973T>C	p.Leu658Pro	1	na		YWTD-EGF	possibly	Tolerated	Neutral	possibly not (0.32)	benign
exon 13	c.1975A>C	p.Thr659Pro	4	yes (2-0)	HdpC at BS	YWTD-EGF	benign	Tolerated	Neutral	yes (-2.04)	benign
exon14	c.2094C>G	p.Cys698Trp	1	na	S-S bond disrupted	Extracell.	probably	Not Tolerated	Pathological	yes (-2.53)	deleterious
exon14	c.2120A>T	p.Asp707Val	1	na		YWTD-EGF	probably	Not Tolerated	Pathological	yes (-3.00)	deleterious
exon14	c.2132G>C	p.Cys711Ser	2	na	S-S bond disrupted	YWTD-EGF	probably	Not Tolerated	Pathological	yes (-2.32)	deleterious
exon 14	c.2140G>C	p.Glu714Gln	1	na	CC at ES	Extracell.	benign	Tolerated	Neutral	no (0.85)	benign
exon17	c.2482T>C	p.Tyr828His	1	na		Cytoplasm.	probably	Not Tolerated	Neutral	possibly (-0.09)	deleterious

Prediction of damaging effect at the protein level performed with Polyphen (http://genetics.bwh.harvard.edu/pph), SIFT (http://sift.jcvi.org), Pmut (http://mmb2.pcb.ub.es/pmut) and SNP3D (http://www.snps3d.org) softwares.

na: not available.

* nb of affected carriers - nb of unaffected non carriers. S-S: Disulfide. LB: Ligand Binding. BS: Buried Site. ES: Exposed Site. HdpC: Hydrophobicity Change. CC: Charge Change. The reference sequences used for *LDLR* were P01130.1 (SwissProt) orNP_000518.1 (NCBI RefSeq). Underlined: prediction different from the three others Bold: prediction of damaging effect was similar with either Polyphen, SIFT, Pmut or SNPs3D.

#### New mutational events leading to abnormal protein size

All nonsense mutations (9) and frameshift variations (41) were deemed as FH-causing mutations, since their theoretical consequence is the synthesis of a truncated protein (Table 1). Prediction of the damaging effect remained difficult for the 19 major rearrangements detected by MLPA since the exact breakpoints were not investigated (Table 1). The main mechanism reported to explain large deletions or duplications is homologous recombination between *Alu* sequences that are numerous in *LDLR* (Lehrman et al. 1987). Only introns 9 and 13 do not contain *Alu* sequences and no major deletion or duplication involving one of these two introns has been reported to date. In the 1990s, deletion breakpoints were sequenced in 14 of the 39 deletions reported in *LDLR*, and 12 involved an *Alu* repeat at both endpoints (Hobbs et al. 1992, Nissen et al. 2006). FH Potenza is a 5 kb deletion that joins a coding sequence in exon 13 to an *Alu* repetitive element in intron 15 (Lehrman et al. 1986). FH Helsinki is a 9.5 kb deletion that does not involve *Alu* sequences at either end of the deletion (Aalto-Setälä et al. 1989). Except for these two examples, data indicate that large deletions or duplications are mainly due to homologous recombination between two *Alu* sequences located in deep intronic sequences, far from splice sites. Therefore, in accordance with this observation and with respect to the translation frame of *LDLR* exons, protein variants could be predicted (Table 1).

#### New intronic variations and small in frame deletions, insertions, or indels

The majority of FH-causing variations within *LDLR* have been investigated at the DNA level, but only a small number of these were corroborated by cellular functional studies. From these few studies and from *in silico* analyses, it is now possible to predict the damaging effect at the protein level. The putative causal effect of each new event was also estimated through Familial Segregation Analysis (FSA) when available.

From the 20 new intronic variations, 10 (50%) were predicted to be deleterious by NetGene2 and NNSPLICE predictor tools, and this could be supported by FSA in three pedigrees. Six (30%) were predicted to be benign with both tools. Surprisingly, the only one for which FSA could be performed revealed the presence of the c.941-12G>A variation in the three affected members analyzed (Table 2). Furthermore, RT-PCR analysis of monocyte mRNA showed an abnormal splicing of intron 6 (data not shown). Four (20%) intronic variations were predicted to be deleterious by only one of the two tools (Table 2).

The 10 in frame del/ins were predicted to be benign, except c.667_693del27bp that was predicted to create a new donor splice site 59 bp downstream with NNSPLICE (Table 2). FSA could be performed for three families, thus indicating that even if predicted to be benign, the familial inheritance of these variations suggested causality. Interestingly, the silent variation p.Leu605Leu was predicted to create a new donor site at position 1813 with a predicted score at 0.58 when the physiological one remains at 0.50 (NNSPLICE). This new donor splice site could lead to: the substitution of p.Leu605 by a threonine, the deletion of 11 amino acids, a frameshift, and a premature termination 49 codons downstream. Furthermore, FSA showed that p.Leu605Leu was carried by the 4 affected family members and not by the unaffected, thus supporting causality. The use of RT-PCR analysis of *LDLR* mRNA from isolated blood cells is necessary to support this point as has been shown for another silent mutation, p.Arg406Arg (Bourbon et al. 2007).

#### New missense variations

Seventy new missense variations were detected here (Table 3). For 28 substitutions, prediction of a damaging effect was similar with either Polyphen, SIFT, Pmut or SNPs3D. For 36 variations, only one prediction was different from the three others and was not always given by the same software (underlined in Table 3). Finally, 6 missense variations (“?” last column, Table 3) were predicted neutral twice and pathogenic twice. Altogether, these analyses showed that 51 (73%) of the new missense variations were very probably deleterious, whereas 13 (19%) were very probably benign. Interestingly, the missense variation c.2140G>C (p.Glu714Gln) was predicted to be benign in Table 3, but to create the loss of the intron 14 donor splice site in Table 2.

#### New promotor variations

Four new DNA variations were found in the promoter sequence: c.-140C>T, c.-155_-150 delACCCCAinsTT, c.-219dupA and c.-267A>G. The first two fall within sterol regulatory elements, SRE1 (-130 to -144) and SRE2 (-145 to -161), respectively (Südhof et al. 1987; Liu J et al. 2000). The third one falls within a cis-acting element FP1 (-219 to -238) (Mehta et al. 1996). The last one falls close to the 3'end of FP2 (-268 to -280).

In conclusion, 78% (136/175) of the new molecular events identified in *LDLR* were very probably FH-causing mutations and were present in 79% (176/222) probands, whereas 16% (28/175) were very probably benign and were present in 16% (35/222) of probands, suggesting that the ADH-causing mutation remains to be identified in this last group. Altogether, these observations confirm the care needed in the interpretation of novel sequence variants and the relevance of functional analysis. Moreover, these results underscore the care needed in the overall interpretation of *in silico* predictions, FSA and *in vitro* functional studies.

### Variations in *APOB, PCSK9* and other genes

The *APOB*-p.Arg3527Gln mutation was identified in 89 probands and 10 mutations in *PCSK9* were found (Abifadel 2003, Allard 2005, Abifadel 2010 personal data). The respective contribution of each gene to ADH was 73.9% *LDLR*, 6.6% *APOB*, 0.7% *PCSK9* and 19.0% “Others”. The identification of this “Others” group of ADH patients clearly demonstrates that there is at least one other disease gene involved in ADH. Furthermore, because of the numerous proteins involved in cholesterol homeostasis, this new group of patients is very probably a heterogeneous class of molecular defects. This is supported by the identification of the *LDLRAP1* gene (also known as *ARH*) which encodes a protein required for clathrin-mediated internalization of the LDL receptor by liver cells (Garcia et al. 2001), but also by our recent report of the localization of a new ADH gene at 16q22.1 (Marques et al. 2010). The percentage of this new group of ADH reported here (19%) is in the range of previously reported large cohort studies that estimated it between 12% and 48% (Varret et al. 2008).

### Clinical and biological features of subjects from the four molecular groups

The four molecular groups were named FH, FDB, PCSK9 and «Others» for carrying a mutation in *LDLR, APOB, PCSK9* and other genes, respectively. The four groups were composed of 190F/192M, 43F/12M, 4F/6M and 30F/21M, respectively, showing a significant difference in the sex ratio between FH/FDB, FDB/«Others», and PCSK9/«Others». Significant differences in the ages of patients were also observed across the four groups. «Others” [median of 46 years old, range: 9-78 (N=51)] were significantly older than FH [median of 37 years old, range: 2-64 (N=382)] (p=0.002), FDB [median of 37 years old, range: 5-61 (N=55)] (p=0.047), and PCSK9 [median of 38 years old, range: 3-49 (N=10)] (p=0.042). To overcome these differences for age and sex of patients among the four groups, we adjusted lipid values for age and gender of a reference French population and expressed them as multiples of median (MoM).

Total and LDL cholesterol levels were significantly higher for FH and PCSK9 when compared to FDB and «Others», and for FDB when compared to «Others” (Supp. Figure S1, panels A and B). As expected, no significant differences were observed for HDL cholesterol levels between FH, FDB and PCSK9 (Supp. Figure S1, panel C). Interestingly, HDL cholesterol levels were significantly higher for «Others” when compared to FH and PCSK9. Triglycerides levels were significantly higher for FH than FDB, as previously reported (Miserez and Keller 1995; Ejarque et al. 2008), and for «Others” when compared to FDB or PCSK9 (Supp. Figure S1, panel D).

Frequency of tendon xanthomas was significantly different only between FH and «Others», with 57% (70/123) and 14% (5/35), respectively (p<0.0001). Frequency of evidence of CHD was also significantly different between FH and «Others», with 68% (44/65) and 41% (11/27), respectively (p<0.016).

Based on the results presented here, a gradient of severity could be drawn for ADH: FH = PCSK9 > FDB > «Others». «Others” seemed to be the less severe group with total and LDL-cholesterol levels significantly lower and presence of xanthomas or evidence of CHD rarer. Furthermore, the age of probands was higher, thus suggesting that it may be diagnosed later in life. Another feature of «Others” was an HDL-cholesterol level significantly higher that should be protective against CHD. This observation could explain the lower frequency of CHD in this group when compared to FH.

## CONCLUSION

In conclusion, mutations in *LDLR* remain the main cause of ADH, and their already large spectrum is here widened with the report of 175 new sequence variations. We also demonstrated the specificity of the spectrum of *LDLR* gene mutations in the French population when compared to other countries, thus underscoring the requirement of specific national molecular screening strategies. More than ¾ of these variations likely cause familial hypercholesterolemia as inferred from the predicted effect on structure and 16% are probably benign, with the remainder requiring careful interpretation and further functional analyses to avoid a false positive diagnosis. Although it had been stated that most human ADH mutations in *LDLR* and other genes had been documented, the relatively high number of new mutations reported here suggests that a substantial proportion of mutations across all human communities remains unidentified.

This is the largest French ADH cohort ever reported and it allowed statistical analysis of clinical and biochemical data. The comparison of the four molecular groups showed, for the first time, that a significant gradient of severity could be established for ADH: FH = PCSK9 > FDB > «Others». Finally, we report a more precise estimation of the percentage of ADH non*LDLR*/non*APOB*/non*PCSK9* patients that is close to 19%.

## Figures and Tables

**Supp. Table S1:** Compared distribution of each type of mutation in the *LDLR* gene between the French cohort and worldwide reports*

	% in the French cohort (All probands)	% in the French cohort (Unique events)	% in worldwide reported unique events*	p value **
Missense	47.7	46.0	47	0.793
Nonsense	17.8	11.3	9	0.143
Frameshift	11.8	14.6	19	0.053
In frame deletions, insertions or indels	2.7	3.8	4	0.743
Splice	11.2	13.6	8	0.002
5′ UTR	0.4	1.0	2	0.130
Major rearangements	8.5	9.7	11	0.491

N	1012	391	1066	

* Leigh et al.2008.

** Chi2 test.
